# Determining the Most Important Physiological and Agronomic Traits Contributing to Maize Grain Yield through Machine Learning Algorithms: A New Avenue in Intelligent Agriculture

**DOI:** 10.1371/journal.pone.0097288

**Published:** 2014-05-15

**Authors:** Avat Shekoofa, Yahya Emam, Navid Shekoufa, Mansour Ebrahimi, Esmaeil Ebrahimie

**Affiliations:** 1 Department of Crop Science, North Carolina State University, Raleigh, North Carolina, United States of America; 2 Department of Crop Production and Plant Breeding, Shiraz University, Shiraz, Iran; 3 Department of Computer Engineering and Information Technology, Amirkabir University of Technology, Tehran, Iran; 4 Department of Biology, School of Basic Sciences, University of Qom, Qom, Iran; 5 School of Molecular and Biomedical Science, The University of Adelaide, Adelaide, Australia; Swiss Federal Institute of Technology (ETH Zurich), Switzerland

## Abstract

Prediction is an attempt to accurately forecast the outcome of a specific situation while using input information obtained from a set of variables that potentially describe the situation. They can be used to project physiological and agronomic processes; regarding this fact, agronomic traits such as yield can be affected by a large number of variables. In this study, we analyzed a large number of physiological and agronomic traits by screening, clustering, and decision tree models to select the most relevant factors for the prospect of accurately increasing maize grain yield. Decision tree models (with nearly the same performance evaluation) were the most useful tools in understanding the underlying relationships in physiological and agronomic features for selecting the most important and relevant traits (sowing date-location, kernel number per ear, maximum water content, kernel weight, and season duration) corresponding to the maize grain yield. In particular, decision tree generated by C&RT algorithm was the best model for yield prediction based on physiological and agronomical traits which can be extensively employed in future breeding programs. No significant differences in the decision tree models were found when feature selection filtering on data were used, but positive feature selection effect observed in clustering models. Finally, the results showed that the proposed model techniques are useful tools for crop physiologists to search through large datasets seeking patterns for the physiological and agronomic factors, and may assist the selection of the most important traits for the individual site and field. In particular, decision tree models are method of choice with the capability of illustrating different pathways of yield increase in breeding programs, governed by their hierarchy structure of feature ranking as well as pattern discovery via various combinations of features.

## Introduction

Agriculture is an information-intensive industry from an essential point of view. Many factors such as sowing date, soil type, fertilizer, location, hybrid, season duration, etc. influence yield and yield components of a grain crop and they are well needed by agricultural experts [1]. Exploring the agricultural technologies of traits related to the control of crop grain yield reductions has a poor record of application [2]. Furthermore, experimental studies remain at an empirical level in which observational evidence is sought for yield increase by genotypes under limited spatial and temporal tests. The utility of these results is limited because there is usually considerable genotype × environment interaction [3].

For example, maize (*Zea mays* L.) yield is a function of the number harvested kernels per unit land area and the individual kernel weight (KW). Kernel weight and its development show a wide variability due to genotype, environment, crop management, and all possible interactions. Commercial maize hybrids differ markedly in the patterns (rate and duration of kernel growth) behind differences in final KW [4], [5], [6].

Some research thus expects to build an intelligent agricultural information system to assist experts and to help improve agricultural technologies [1]. Recently, agricultural and biological research studies have used various techniques of data mining for analyzing large data sets and establishing useful classification patterns within these data sets [7]. However, data mining methods are still expected to bring more fruitful results [1], [7], [8].

Recently, intelligent data mining and knowledge discovery by artificial neural network, decision trees, and feature selection algorithms have become the important revolutionary issues in prediction and modeling [8], [9], [10], [11], [12], [13], [14]. Data mining problems often involve hundreds or even thousands of variables [15].

Machine learning methods have three main steps. The first step is extracting/collecting the n-dimensional features vector in order to reflect different aspects of the conditions (features) with a class label attached. The second step of machine learning approach is application of machine learning method (or classifier) for prediction of the class label of the features input. Currently, many machine learning methods, such as neural networks, support vector machine (SVM), and decision trees have been successfully developed. Each algorithm may be run with different criteria and they have been widely employed in many scientific fields, including biological systems. The main role of these systems is to predict unknown situations based on some known features and their efficiencies have already been proven by many publications. The third step is measuring the performance of the prediction method and its validity using approaches such cross validation technique and independent evaluation (IE) datasets.

Fitting a neural network or a decision tree to a set of variables this large may require more time than is practical [16]. As a result, the majority of time and effort spent in the model-building process involves determining which variables to include in the model. Feature selection allows the variable set to be reduced in size, creating a more manageable set of attributes for modeling [12], [13], [14], [17], [18].

The decision tree algorithm [19] predicts the value of a discrete dependent variable with a finite set from the values of a set of independent variables. As a popular data-mining method, the decision tree algorithm is superior to other algorithms in many aspects. It is computationally fast, makes no assumption on data distribution, can attain nonlinear mapping and easily interpretable rules, and has an embedded ability for feature selection [20]. A decision tree is constructed by looking for regularities in data, determining the features to add at the next level of the tree using an entropy calculation, and then choosing the feature that minimizes the entropy impurity [12], [13]. Decision tree is method of choice for prediction since it presents hierarchical ranking of important features and provides a clear image of effective factors [12], [13].

Herein, we used various clustering, screening, and decision tree models to determine the most important features responsible for increasing maize grain yield between all available features. Understanding the importance of features and relationship of maize field conditions traits (features) provides a comprehensive view about data mining and maize grain yield.

## Results

Various traits (features) which may play key roles in determining maize grain yield are presented in Table 1. In this study, a wide range of modeling algorithms was applied on a dataset of these features to determine the most important features of maize grain physiology.

**Table 1:** Traits involved in maize grain yield based on literature.

Type of treatment	Country	Authors reference	Sophisticated randomization layouts*
Defoliation, plant densities, hybrids	Iran	[21]	RCBD/split-split plot arrangement
Defoliation, Restricted pollination	Argentina	[22]	RCBD
Hybrids	Argentina	[23]	RCBD
Plant densities, hybrids	Argentina	[24]	RCBD
Hybrids	India	[25]	RCBD/split plot arrangement
Plant densities, Restricted pollination, hybrids	USA	[26]	strip plots
Hybrids, nitrogen levels	Argentina	[27]	RCBD included a combination of three factors
Defoliation, kernel removal	USA	[28]	RCBD
Hybrids	Canada	[29]	RCBD
Plant densities, Restricted pollination, hybrids	USA	[30]	RCBD
Shading, thinning, hybrids	Argentina	[31]	RCBD
Hybrids	USA	[32]	RCBD

^*^RCBD: Randomized Complete Block Design.

### Screening Models

#### Feature Selection

Features classification (Table 2) indicated that among tested features, 12 features were the most important traits related to maize grain yield (Table 2). These included sowing date-location (country), stem dry weight, soil type, P applied, kernel number per ear, final kernel weight, soil type, season duration, soil pH with 1.0 value, and maximum kernel water content (0.999 value), N applied (0.985 value), and cob dry weight (0.980 value). The days to silking feature (0.926) was recognized to have a marginal effect on maize grain yield.

**Table 2:** The most important features involved in maize grain yield, selected by feature selection.

Rank	Field	Type	Importance	Value*
1	Sowing date-country (days)	Set	Important	1.0
2	Stem dry weight (g plant^−1^)	range	Important	1.0
3	Soil type	Set	Important	1.0
4	P applied (kg ha^−1^)	range	Important	1.0
5	Kernel number per ear	range	Important	1.0
6	Final kernel weight (mg)	range	Important	1.0
7	Season duration (days)	range	Important	1.0
8	Soil pH	range	Important	1.0
9	Maximum kernel water content (mg)	range	Important	0.999
10	N applied (kg ha^−1^)	range	Important	0.985
11	Cob dry weight (g plant^−1^)	range	Important	0.980
12	Days to silking	range	Marginal	0.926
13	Density (kg ha^−1^)	range	Unimportant	0.848
14	Hybrids type	Set	Unimportant	0.836
15	Kernel dry weight (mg)	range	Unimportant	0.702
16	Kernel growth rate (mg °C day^−1^)	range	Unimportant	0.651
17	Duration of the grain filling period (°C day)	range	Important	0.622
18	Defoliation	Set	Unimportant	0.413
19	Leaf dry weight (g plant^−1^)	range	Unimportant	0.299
20	Day (time of defoliation applied)	range	Unimportant	0.294
21	K applied ( kg ha^−1^)	range	Unimportant	0.113

^*^Values closer to 1 show the higher importance.

The rest of features [plant density (0.848 value), hybrid type (0.836 value), kernel dry weight (0.702 value), kernel growth rate (0.651 value), duration of the grain filling period (0.622 value), defoliation (0.413 value), leaf dry weight (0.299 value), day (time of defoliation applied) (0.294) and K applied (0.113 value)] revealed to be unimportant features. We found that the classifier performance improved by eliminating redundant features (Table 2).

In our study, redundant features were plant density (plant ha^−1^), hybrid type, kernel dry weight (mg), kernel growth rate (mg °C day^−1^), duration of the grain filling period (°C day), defoliation, leaf dry weight (g plant^−1^), day (time of defoliation applied) and K applied (kg ha^−1^) (Table 2).

#### Anomaly detection model

When the anomaly detection model was applied on dataset with feature selection criteria, the records divided into two peer groups with an anomaly index cutoff of 1.801, no record in the first peer group and one record in the second peer group found to be anomaly. The counts of mean kernel weight, defoliation, duration of grain filling period with average indices of 0.237, 0.214 and 0.124, respectively, occurred in each anomalous record.

The same peer groups with the same number of record in each group and the same number of anomalous records found when the model applied on the dataset without feature selection, but the count of mean kernel weight, defoliation, duration of grain filling period with average indices of 0.222, 0.201 and 0.117, respectively, were the three traits contributed to each anomalous record.

### Clustering Models

#### K-Means

**Figure 1:**
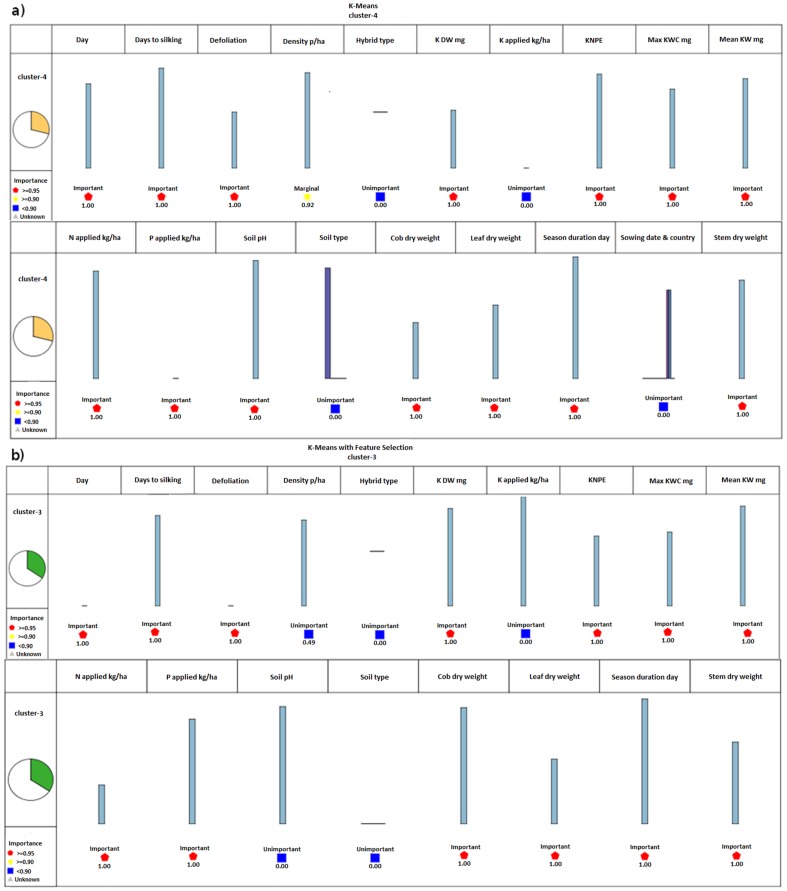
Comparison of the filtering of dataset with feature selection algorithm based on K-Means model. (a) Most important generated cluster without feature selection filtering, cluster 4. (b) Most important generated cluster with feature selection filtering, cluster 3. 3. When K-Means model was applied on data filtered with feature selection, the records were put into 5 groups or clusters. When the model was applied on dataset without feature selection filtering, again five clusters were generated. In this clustering model, more than 28% of the records were put into the fourth cluster when the K-Means model was applied on the dataset without feature selection (Fig. 1a). When the K-Means model was applied on the dataset with feature selection filtering, more than 34% of the records were put into the third cluster (Fig. 1b). The number of iteration declined from 5 to 4 when feature selection applied on dataset.

When K-Means model was applied on data filtered with feature selection, the records were put into 5 groups or clusters (18, 5, 43, 23 and 36 records in each cluster, respectively). When the model was applied on dataset without feature selection filtering, again five clusters with 18, 5, 34, 36 and 32 records were generated. In this clustering model, more than 28% of the records were put into the fourth cluster when the K-Means model was applied on the dataset without feature selection, respectively (Fig. 1a). When the K-Means model was applied on the dataset with feature selection filtering, more than 34% of the records were put into the third cluster (Fig. 1b). The number of iteration declined from 5 to 4 when feature selection applied on dataset.

### Decision Tree Models

#### Classification and regression tree (C&RT)

**Figure 2:**
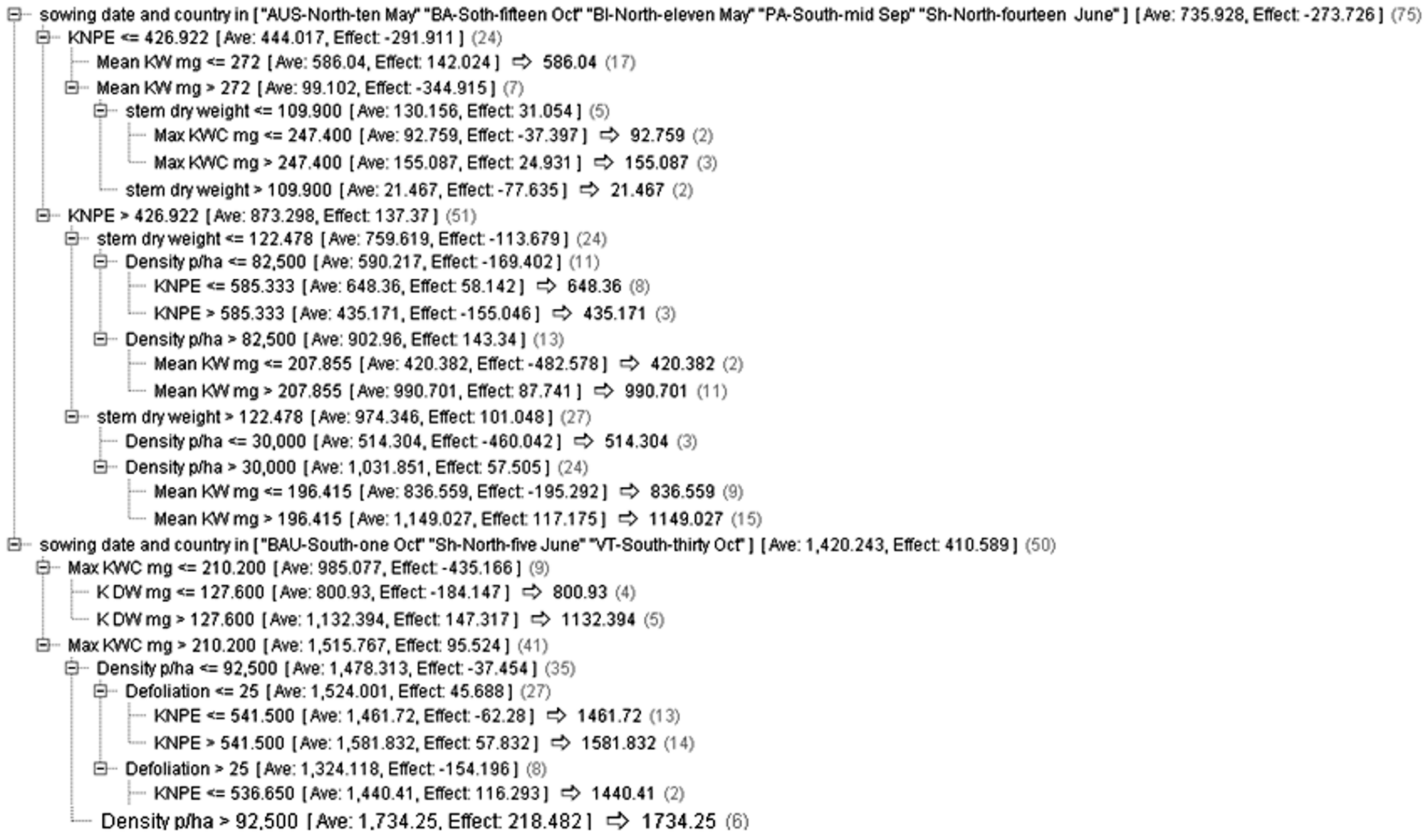
Decision tree generated by C&RT model run on dataset with feature selection filtering. This model suggests that the following 3 combination of features (routes) can result in high maize grain yield: (1) Sowing date and country in [“AUS-North-ten May” “BA-South-fifteen Oct” “BI-North-eleven May” “PA-South-mid Sep” “Sh-North-fourteen June”] and KNPE >426 and Stem dry weight >122.478 and Mean KW >196.4 mg, (2) Sowing date and country in [“BAU-South-one Oct” “Sh-North-five June” “VT-South-thirty Oct”] and Max KWC >210.2 mg and KNPE >541, and (3) Sowing date and country in [“BAU-South-one Oct” “Sh-North-five June” “VT-South-thirty Oct”] and Max KWC >210.2 mg and Density p/ha>92500.

In the C&RT node, a tree with a depth of five was created with the most important feature used to build the tree being the sowing date-country {part one included as [AUS-N-10 May (Ames-IA-USA, North, 10 May), BA-S-15 Oct (INTA-Balcarce-Buenos Aires-Argentina, South, 15 Oct), BI-N-11 May (Bruner-Iowa Stat University-Ames, North, 11 May), PA-S-Mid Sep (INTA-Parana-Argentina, South, Mid Sep), and Sh-N-14 June (Badjgah-Shiraz-Iran, North, 14 June)] and part two [BAU-S-1 Oct (Department of Plant Production at the University of Buenos Aires, South, 1 Oct), Sh-N-5 June (Badjgah-Shiraz-Iran, North, 5 June), and VT-S-30 Oct. (Experimental Field of Nidera Argentina S.A. in Venado Tuerto, South, 30 Oct.)]}. The same results were obtained when feature selection was selected Fig. 2.

#### CHAID and Exhaustive CHAID

**Figure 3:**
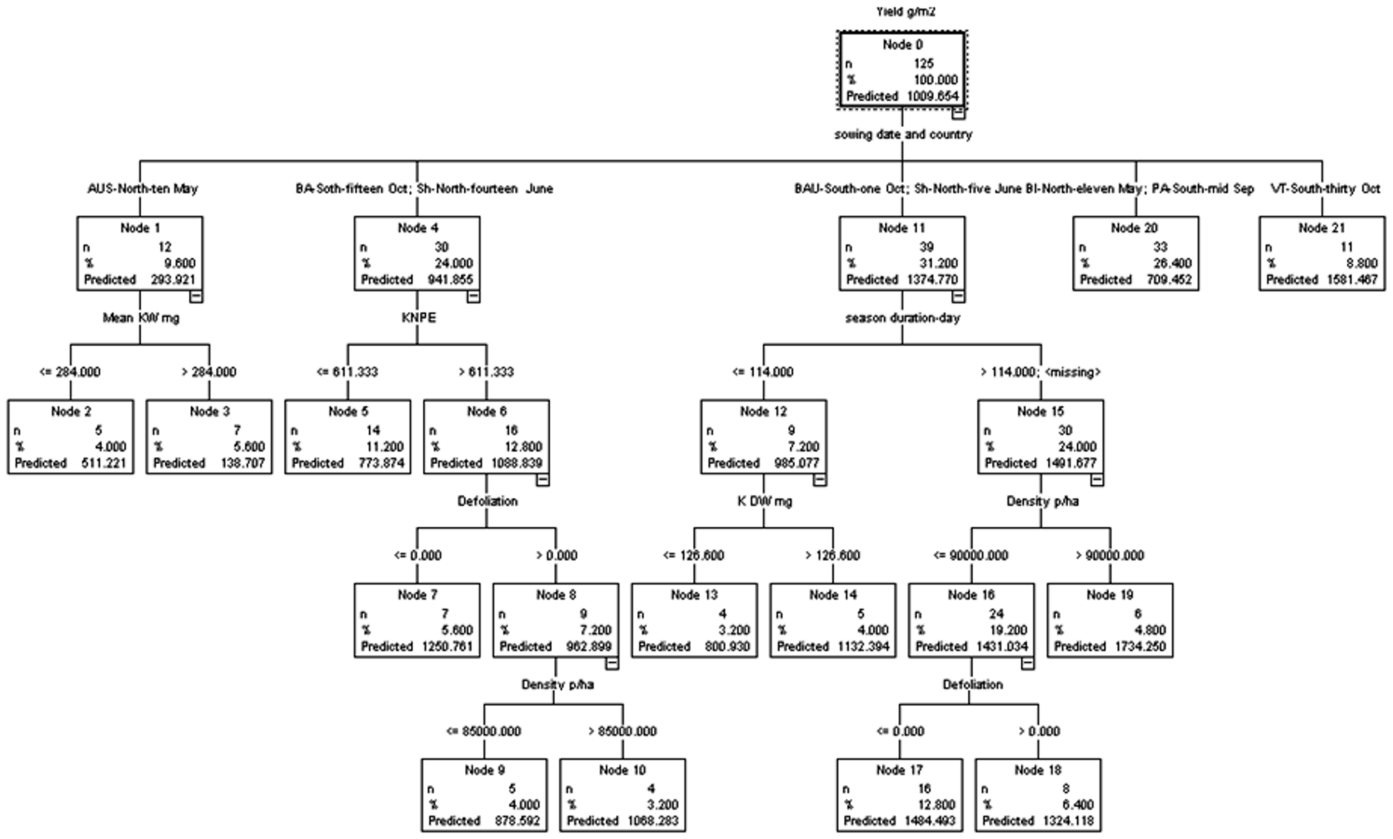
Decision tree generated by CHID algorithm run on dataset with feature selection filtering. This tree presents hierarchy structure of traits (features). Different combinations of features such as “Sowing date and country”, “Season duration”, “Density”, “Defoliation”, KNPE, and “Mean KW” significantly alter maize yield.

When the CHAID model was applied to the data with or without feature selection, a tree with a depth of 4 was generated. The sowing date-country was the main attribute to build the four branches. If this feature was equal to AUS-N-10 May (Ames-IA-USA, North, 10 May), the mean KW mg was the most important trait related to the depth one and maize grain yield (Fig. 3). If the sowing date-country was equal to [BA-S-15 Oct (INTA-Balcarce-Buenos Aires-Argentina, South, 15 Oct), and Sh-N-14 June (Badjgah-Shiraz-Iran, North, 14 June)] the kernel number per ear (KNPE) was the important feature. If the value of KNPE was more than 611.3, defoliation was the most related feature to the depth two; sowing date-country (Fig. 3).

The same trees with the same features and values were generated when exhaustive CHAID model applied to datasets with or without feature selection filtering.

## Discussion

Here, for the first time, we applied different data mining models to study different fields in respect to 22 physiological and agronomic traits (features) attributed to maize grain yield. We analyzed the performance of different screening, clustering, and decision tree modeling on the dataset with or without feature selection filtering for discriminating important and unimportant traits as well as finding pathways of factor combinations which result in high yield.

Regarding the fact that agricultural traits such as yield can be affected by a large number of diverse factors (features), different pattern recognition algorithms have a great potential of use to highlight the most important factors and illustrate the different combination of factors which result in high/low yield outcome based on their pattern recognition capacity. In comparison to the common univariate and multivariate based methods in agriculture, the application of the presented machine learning based methods in this study enables more complex data to be analyzed, particularly when the feature space is complex and all data do not follow the same distribution pattern [18], [35], [36]. In fact, novel data mining approaches can be seen as an extension/improvement of previous multivariate based methods when the number of factors (columns) and the number of cases (rows) increases.

We expect recent data mining technologies to bring more fruitful results, particularly under the following circumstances: (1) when data present an important number of traits (features) with missing values due to the capability of data mining approaches in dealing with missing data; (2) when not only the yearly yield data, but also extended data in long time period and in different locations is reported.

The sowing date-location (country) ranked as the most important feature, and it was used in decision tree models to create the main subgroups and branches (Fig. 2 and Fig. 3). The relationship between one important management decision, planting date, and maize yield potential has been previously documented by Lauer *et al.* [37] and Nielsen *et al.* [38]. Our findings were also in line with previous studies, which have shown that grain yield is closely related to the number of kernels that reach maturity and kernel weight (KW) [4], [6], [39].

The number of peer groups (two groups), and also the anomaly index cut off did not change when feature selection applied on the dataset. Although the number of clusters generated by K-Means modeling did not change between the models with or without feature selection, the number of iteration declined from 5 to 4, showing the positive effects of feature selection filtering on removing outliers.

**Figure 4:**
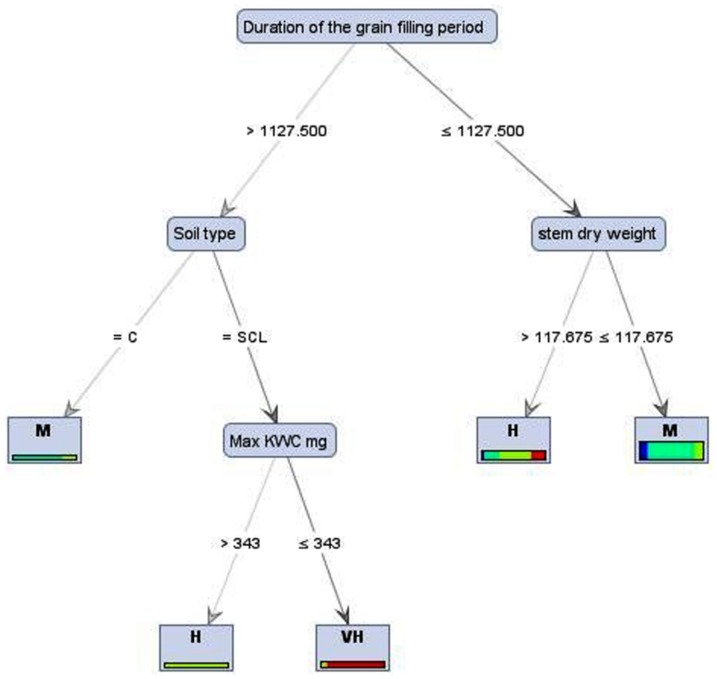
Tree induced by decision tree algorithm with information gain ratio (L: less than 500 maize grain yield g m^−2^, M: 501–1000 maize grain yield g m^−2^, H: 1001–1500 maize grain yield g m^−2^, VH: more than 1500 maize grain yield g m^−2^, C: Clay, sandy clay). The tree shows that there is 2 pathways (routes) for reaching high yield according to this model (1) When “Duration of the grain filling period”>1127.5 and “Soil type” is Sandy clay, and (2) When “Duration of the grain filling period”>1127.5 and “stem dry weight”>117.675.

Results of the best and the worst performances gained when tree induced by decision tree algorithms on the continuous target (output) (Fig. 2 and Fig. 3) and categorical one (Fig. 4), respectively. Generally decision tree algorithms provide a very useful tool to manipulate huge data [20]. In this study, we observed decision tree algorithms run on data with the continuous targets (output) are more acceptable than the categorical target. The findings also confirm that the types and the distributions of dataset in continuous target are different from the categorical one; therefore using decision tree algorithms on the continuous target (e.g., maize grain yield) may be seen as a suitable candidate for crop physiology studies. These results are in general agreement with previous evidence [40]. Within decision tree models, C&RT algorithm was the best for yield prediction in maize based on physiological and agronomical traits which can be employed in future breeding programs.

One of the major advantages of the mentioned machine learning techniques for crop physiologists/plant breeders is the possibility to search throughput large datasets in order to discover patterns of physiological and agronomic factors. In particular, decision tree models are strong in pattern recognition and rule discovery by simultaneous looking a combination of factors in respect to yield, instead on analysing each feature (trait) separately. As example, C&RT decision tree model run on dataset with feature selection filtering (Figure 2) suggests that the following 3 combination of features (routes) can result in high maize grain yield:

Pathway1: Sowing date and country in [“AUS-North-ten May” “BA-South-fifteen Oct” “BI-North-eleven May” “PA-South-mid Sep” “Sh-North-fourteen June”] and KNPE >426 and Stem dry weight >122.478 and Mean KW >196.4 mg.

Pathway 2: Sowing date and country in [“BAU-South-one Oct” “Sh-North-five June” “VT-South-thirty Oct”] and Max KWC >210.2 mg and KNPE >541.

Pathway 3: Sowing date and country in [“BAU-South-one Oct” “Sh-North-five June” “VT-South-thirty Oct”] and Max KWC >210.2 mg and Density p/ha>92500.

In other words, the discovered patterns in machine learning methods can be seen in some ways as extension of interaction and factorial experiments in the traditional statistical designs in agriculture but in larger scale.

Another strength of decision tree models, which has a great potential use in agriculture, is its hierarchy structure. In a decision tree, the features which are in the top of tree such as “Sowing date and country” in decision tree generated by C&RT model (Figure 2) or “Duration of the grain filling period” at decision tree with information gain ratio (Figure 4) have more influences/impact in determining the general pattern in data, compared to the features in the branches of tree. Another example, in C&RT model (Figure 2), KNPE sits on the above of Mean/Max KW and has more contribution in dimension of target variable (maize yield) and possibly higher influence than Mean/Max KW.

This topography/hierarchy structure of data in relation to target variable (yield) cannot be obtained from the current classical methods of analysis agricultural experiments whereas decision tree opens a new avenue in this field.

As a pioneer study, this work opens a new avenue to encourage the other researchers to employ novel data mining approaches in their studies. Remarkably, the presented machine learning methods provide the opportunity of considering an unlimited wide range for each feature as well as an unlimited number of features. Increasing the number and the range of features (variables) in future data mining studies can lead to achieving more comprehensive view where this view is hard to be obtained from the separated small scale experiments. Recent progress in machine learning packages such as RapidMiner (http://rapidminer.com/, Dortmund, Germany) and SPSS Clementine (http://spss-clementine.software.informer.com/, USA), which offer a user friendly environment, provides this opportunity for the general agronomist/biologist (without the knowledge of software programing) to easily run and employ the selected data mining models without any difficulty.

In conclusion, agriculture is a complex activity which is under the influences of various environmental and genetic factors. We suggest that novel data mining methods have the great potential to deal with this complexity. Two characteristics of data mining methods have the great potential of employment in agriculture and plant breeding: (1) feature selection (attribute weighting) algorithms to distinguish the most important features within many factors and (2) pattern recognition algorithms such as decision tree models to shed light on various pathways toward of yield increase based on factor combination.

## Methods

### Data collection

Data presented in this study was collected from the two sources: (1) two field experiments, and (2) literature on the subject of maize physiology (Table 1, Table S1).

#### Data collection – field experiments

Data were obtained from two carried out experiments without any discernible nutrient or water limitations during 2008 and 2009 growing seasons, at the Experimental Farm of the College of Agriculture, Shiraz University, Badjgah, [29° 50′ N and 52° 46′ E; elevation 1810 m above mean sea level (ASL)] by the authors. The experimental design was a randomized complete block design (RCBD) with three replicates and treatments in a designed split-split plot arrangement. Three hybrids (370, Maxima 524, and 704) were the main plots, the plant densities (7.5, 8.5 and 9.5 pl m^−2^) were allocated to subplots, and defoliation (control-without defoliation, 50% of defoliation at 25, and 35 days after silking) in the sub-subplots.

In both experiments, kernel samples were collected at 7 day intervals 10 days after silking until physiological maturity. Samples were taken from the central rows of each plot. The entire ear with surrounding husks was immediately enclosed in an airtight plastic bag and taken to the lab, where 10 kernels were removed from the lower third of each ear. Fresh weight was measured immediately after sampling, and kernel dry weight was determined after drying samples at 70°C for at least 96 h. Kernel water content was calculated as the difference between kernel fresh weight and dry weight. Differences among treatments during grain-filling period (i.e., from silking until physiological maturity) were recorded. Also, growing degree days (GDD) were calculated starting at silking using mean daily air temperature with a base temperature of 10°C. Kernel growth rate during the effective grain-filling period was determined for each hybrid at each year by fitting a linear model [Eqs. (1)]: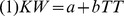 where, TT is thermal time after silking (in °Cd), a is the Y-intercept (in °Cd), and b is the kernel growth rate during the effective grain-filling period (in mg °Cd^−1^). The linear model was fitted to the kernel dry weight data using the iterative optimization technique of Table Curve V 3.0. Daily TT values were obtained with a base temperature of 10°C. Mean daily air temperature was calculated as the average of hourly air temperatures registered at a weather station located at the nearest place to the experimental plots for both years

#### Data collection – literature

The reference papers are listed in Table 1 and Table S1. The original sophisticated randomization layouts of these experiments (RCD, RCBD, etc.) are presented at Table 1.

As a result, 166 records (rows) with 22 traits (features/columns) including kernel number per ear, nitrogen (N) fertilizer applied (kg ha^−1^), plant density (plant ha^−1^), sowing date-location (country), stem dry weight (g plant^−1^), kernel dry weight (mg), duration of the grain filling period (°C day), kernel growth rate (mg °C day^−1^), Phosphorous (P) fertilizer applied (kg ha^−1^), mean kernel weight (mg), grain yield (g m^−2^), season duration (days), days to silking, leaf dry weight (g plant^−1^), mean kernel weight (mg), cob dry weight (g plant^−1^), soil pH, potassium (K) fertilizer applied (kg ha^−1^), hybrid type, defoliation, soil type, and the maximum kernel water content (MKWC) (mg) were recorded. The yield was set as the output variable and the rest of variables as input variables. The final data set, prepared for running machine learning algorithms, is presented as Table S1.

### Models

When the target value was continuous, *p* values based on the F statistic were used. If some predictors are continuous and some are categorical in the dataset, the criterion for continuous predictors is still based on the *p* value from a transformation and that for categorical predictors from the *F* statistic. Predictors are ranked by the following rules: (1) Sort predictors by *p* value in ascending order; (2) If ties occur, follow the rules for breaking ties among all categorical and all continuous predictors separately, then sort these two groups (categorical predictor group and continuous predictor group) by the data file order of their first predictors [33], [34]. A dataset of these features was imported into Clementine software [34] for further analysis. The following models run on pre-processed dataset.

### Screening models

This step removes variables and cases that do not provide useful information for prediction and issues warnings about variables that may not be useful.

#### Anomaly detection model

The goal of anomaly detection is to identify cases that are unusual within data that is seemingly homogeneous. Anomaly detection is an important tool for detecting fraud, network intrusion, and other rare events that may have great significance but are hard to find. This model was used to identify outliers or unusual cases in the data. Unlike other modeling methods that store rules about unusual cases, anomaly detection models store information on what normal behavior looks like. This makes it possible to identify outliers even if they do not conform to any known pattern. While traditional methods of identifying outliers generally examine one or two variables at a time, anomaly detection can examine large numbers of fields to identify clusters or peer groups into which similar records fall. Each record can then be compared to others in its peer group to identify possible anomalies. The further away a case is from the normal center, the more likely it is to be unusual.

#### Feature selection algorithm

The feature selection algorithm was applied to identify the attributes (traits) that have a strong correlation with maize grain yield. The algorithm considers one attribute at a time to determine how well each predictor alone predicts the target variable. The important value for each variable is then calculated as (1 – *p*), where *p* is the value of the appropriate test of association between the candidate predictor and the target variable. The association test for categorized output variables differs from the test for continuous variables. In our study, when the target value was continuous, p values based on the F statistic were used. The idea was to perform a one-way ANOVA F test for each predictor; otherwise, the *p* value was based on the asymptotic t distribution of a transformation of the Pearson correlation coefficient. Other models, such as likelihood-ratio chi-square (which also tests for target-predictor independence), Cramer's V (a measure of association based on Pearson's chi-square statistic), and lambda (a measure of association that reflects the proportional reduction in error when the variable is used to predict the target value) were conducted to check for possible effects of calculation on feature selection criteria. The predictors were then labeled as important, marginal, and unimportant, with values >0.95, between 0.95–0.90, and < 0.90, respectively.

### Clustering models

#### K-Means

The K-Means model can be used to cluster data into distinct groups when groups are unknown. Unlike most learning methods, K-Means models do not use a target field. This type of learning, with no target field, is called unsupervised learning. Instead of trying to predict an outcome, K-Means tries to uncover patterns in the set of input fields. Records are grouped so that records within a group or cluster tend to be similar to each other, whereas records in different groups are dissimilar. K-Means works by defining a set of starting cluster centers derived from the data. It then assigns each record to the cluster to which it is most similar based on the record's input field values. After all cases have been assigned, the cluster centers are updated to reflect the new set of records assigned to each cluster. The records are then checked again to see whether they should be reassigned to a different cluster and the record assignment/cluster iteration process continues until either the maximum number of iterations is reached or the change between one iteration and the next fails to exceed a specified threshold.

### Decision tree models

#### Classification and regression tree (C&RT)

This model uses recursive partitioning to split the training records into segments by minimizing the impurity at each step. A node is considered pure if 100% of cases in the node fall into a specific category of the target field.

#### CHAID

This method generates decision trees using chi-square statistics to identify optimal splits. Unlike the C&RT and QUEST models, CHAID can generate non-binary trees, meaning that some splits can have more than two branches.

#### Exhaustive CHAID

This model is a modification of CHAID that does a more thorough job of examining all possible splits, but it takes longer to compute.

## Supporting Information

Table S1 **Final dataset for running machine learning algorithms including the 166 records (rows) (derived from field and literature experiments) and 22 traits (features/columns).** The traits were kernel number per ear, nitrogen (N) fertilizer applied (kg ha^−1^), plant density (plant ha^−1^), sowing date-location (country), stem dry weight (g plant^−1^), kernel dry weight (mg), duration of the grain filling period (°C day), kernel growth rate (mg °C day^−1^), Phosphorous (P) fertilizer applied (kg ha^−1^), mean kernel weight (mg), grain yield (g m^−2^), season duration (days), days to silking, leaf dry weight (g plant^−1^), mean kernel weight (mg), cob dry weight (g plant^−1^), soil pH, potassium (K) fertilizer applied (kg ha^−1^), hybrid type, defoliation, soil type, and the maximum kernel water content (MKWC) (mg). The yield was set as the output variable and the rest of variables as input (predictor) variables.
(XLS)
